# Effects of *Colletotrichum gloeosporioides* and Poplar Secondary Metabolites on the Composition of Poplar Phyllosphere Microbial Communities

**DOI:** 10.1128/spectrum.04603-22

**Published:** 2023-05-23

**Authors:** Linxuan Zhang, Fanli Meng, Wei Ge, Yue Ren, Hangbin Bao, Chengming Tian

**Affiliations:** a The Key Laboratory for Silviculture and Conservation of Ministry of Education, College of Forestry, Beijing Forestry University, Beijing, China; Connecticut Agricultural Experiment Station

**Keywords:** poplar anthracnose, *Colletotrichum gloeosporioides*, phyllosphere microbial community, secondary metabolites

## Abstract

Poplar anthracnose caused by Colletotrichum gloeosporioides is a common disease affecting poplars globally that causes the destruction and alteration of poplar phyllosphere microbial communities; however, few studies have investigated these communities. Therefore, in this study, three species of poplar with different resistances were investigated to explore the effects of *Colletotrichum gloeosporioides* and poplar secondary metabolites on the composition of poplar phyllosphere microbial communities. Evaluation of the phyllosphere microbial communities before and after inoculation of the poplars with *C. gloeosporioides* revealed that both bacterial and fungal OTUs decreased after inoculation. Among bacteria, the most abundant genera were *Bacillus*, *Plesiomonas*, Pseudomonas, *Rhizobium*, *Cetobacterium*, Streptococcus, *Massilia*, and *Shigella* for all poplar species. Among fungi, the most abundant genera before inoculation were *Cladosporium*, Aspergillus, Fusarium, *Mortierella*, and *Colletotrichum*, while *Colletotrichum* was the main genus after inoculation. The inoculation of pathogens may regulate the phyllosphere microorganisms by affecting the secondary metabolites of plants. We investigated metabolite contents in the phyllosphere before and after the inoculation of the three poplar species, as well as the effects of flavonoids, organic acids, coumarins, and indoles on poplar phyllosphere microbial communities. We speculated that coumarin had the greatest recruitment effect on phyllosphere microorganisms, followed by organic acids through regression analysis. Overall, our results provide a foundation for subsequent screening of antagonistic bacteria and fungi against poplar anthracnose and investigations of the mechanism by which poplar phyllosphere microorganisms are recruited.

**IMPORTANCE** Our findings revealed that the inoculation of *Colletotrichum gloeosporioides* has a greater effect on the fungal community than the bacterial community. In addition, coumarins, organic acids, and flavonoids may have recruitment effects on phyllosphere microorganisms, while indoles may have inhibitory effects on these organisms. These findings may provide the theoretical basis for the prevention and control of poplar anthracnose.

## INTRODUCTION

The genus *Populus* in the family Salicaceae has wide distribution and strong adaptability and is fast growing, which makes it commonly used in the development of artificial forests worldwide (1, 2). The total area of planted poplar has increased to 31.4 million ha worldwide, with 96% of this being in Canada and China (3). According to the results of the Ninth National Forest Resources Inventory, China’s artificial poplar forests cover an area of 7.57 million ha with a stock volume of 546 million m^3^, ranking first in the world (4). With the large-scale planting of poplar, the occurrence of poplar diseases and insect pests is increasing. One of the most serious poplar diseases is poplar anthracnose, which is caused mainly by Colletotrichum gloeosporioides (5). This disease can cause significant economic and ecological losses by causing reduced poplar productivity and even tree death (6, 7).

Different species of poplar have different levels of anthracnose resistance, with Populus × canadensis being resistant, Populus tomentosa being susceptible, and Populus × beijingensis having intermediate resistance (8). To date, most studies of poplar resistance have focused on poplar resistance genes (9) and the direct inhibitory effects of poplar secondary metabolites on pathogenic fungi (8, 10). However, the effects of phyllosphere microorganisms on the resistance of different poplar species to anthracnose have not been thoroughly investigated (11). Poplar anthracnose is a leaf disease; therefore, phyllosphere microbes may impact poplar resistance to *C. gloeosporioides*, and pathogen inoculation may manipulate the phyllosphere microbiome as well.

Phyllosphere microbes consist of epiphytic microbes on the leaf surface and endophytic microbes within the leaf tissue (12). These organisms include bacteria, fungi, archaea, and yeasts, and of these, the number is largest for bacteria (13–15). The phyllosphere is a transient and open living environment; therefore, many factors affect phyllosphere microbial communities (16–18). Insect feeding can significantly increase the abundance of bacterial groups inside leaves, possibly via insect-activated plant defense mechanisms (19). Pathogen inoculation will also change the structure of microbial communities in the leaf zone. For example, phytoplasma inoculation has been shown to affect the endophytic bacterial community in the phyllosphere of Vitis vinifera (20). Secondary metabolites produced by plants can also influence the scale and diversity of phyllosphere microbial communities. Kniskern et al. (21) investigated the effects of two specific plant-specific signaling defense pathways (salicylic acid [SA] and jasmonic acid [JA]) on the interfoliar endophytic and epiphytic bacterial communities of Arabidopsis thaliana. They found that the SA-mediated defense responses in *A. thaliana* reduced the diversity of the interfoliar endophytic bacterial community, while the lack of JA-mediated defense in *A. thaliana* resulted in a higher diversity of the interfoliar epiphytic bacterial community (21).

In this study, the bacterial and fungal communities were analyzed by sequencing the V4 region of the bacterial 16S rRNA gene and the ITS1 region of the fungal internal transcribed spacer (ITS) gene, using the high-throughput Illumina MiSeq system (Illumina, USA) to analyze the microbial communities of healthy and anthracnose-infected leaves of three species of poplar with different levels of anthracnose resistance (healthy *Populus* × *canadensis*, *P.* × *beijingensis*, and *P. tomentosa* [Pc-No, Pb-No, and Pt-No, respectively] and *P.* × *canadensis*, *P.* × *beijingensis*, and *P. tomentosa* inoculated with *C. gloeosporioides* [Pc-Cg, Pb-Cg, and Pt-Cg, respectively]). Changes in bacterial and fungal communities were then investigated to determine if community differences were caused by *C. gloeosporioides* inoculation and to elucidate the relationship between the pathogen and host microbial communities. We also evaluated the secondary metabolites of leaves, focusing on coumarins, indoles, and their derivatives, to determine if they influenced the assemblage of poplar microbial communities.

## RESULTS

### Microbial richness and diversity index.

Sequencing of bacteria in leaf samples of three species of poplar yielded 479,417 pairs of reads, from which 478,442 clean reads were produced after paired-end read quality control and splicing. Each sample produced at least 79,438 clean reads, with an average of 79,740. There were no significant differences in phyllosphere bacterial richness and diversity between Pc-No (operational taxonomic units [OTUs], 1,612.67 ± 92.32; Shannon index, 9.06 ± 0.84; Simpson index, 0.99 ± 0.01) and Pb-No (OTUs, 1,597.67 ± 87.39; Shannon index, 9.20 ± 0.79; Simpson index, 0.99 ± 0.01), but these values were significantly higher than those of Pt-No (OTUs, 1,511.00 ± 85.14; Shannon index, 6.97 ± 1.14; Simpson index, 0.93 ± 0.02). Additionally, the Pc-Cg (OTUs, 1,590.33 ± 109.42; Shannon index, 8.26 ± 0.93; Simpson index, 0.97 ± 0.02) and Pb-Cg (OTUs, 1,362.33 ± 73.92; Shannon index, 5.64 ± 1.05; Simpson index, 0.87 ± 0.03) phyllosphere bacterial richness and diversity were lower than those of untreated samples, while the Pt-Cg (OTUs, 1,484.00 ± 47.29; Shannon index, 8.01 ± 1.26; Simpson index, 0.97 ± 0.01) phyllosphere bacterial diversity values were higher than those of the untreated samples (see Table S1 in the supplemental material).

For fungal samples, a total of 3,449,834 pairs of reads were obtained, from which 3,436,760 clean reads were produced after paired-end read quality control and splicing. Each sample produced at least 432,845 clean reads, with an average of 572,793. There was no significant difference in phyllosphere fungal richness and diversity among Pc-No (OTUs, 996.67 ± 28.45; Shannon index, 7.60 ± 0.58; Simpson index, 0.96 ± 0.02), Pb-No (OTUs, 1,005.00 ± 55.05; Shannon index, 8.12 ± 0.62; Simpson index, 0.99 ± 0.01), and Pt-No (OTUs, 982.33 ± 59.53; Shannon index, 8.03 ± 0.74; Simpson index, 0.99 ± 0.01). However, Pc-Cg (OTUs, 958.00 ± 68.02; Shannon index, 1.47 ± 0.23; Simpson index, 0.27 ± 0.13), Pb-Cg (OTUs, 932.00 ± 43.00; Shannon index, 0.74 ± 0.16; Simpson index, 0.11 ± 0.06), and Pt-Cg (OTUs, 778.00 ± 46.29; Shannon index, 0.35 ± 0.09; Simpson index, 0.06 ± 0.03) phyllosphere fungal richness and diversity values were lower than those before inoculation (Table S2).

### Diversity of microbial composition.

**FIG 1:**
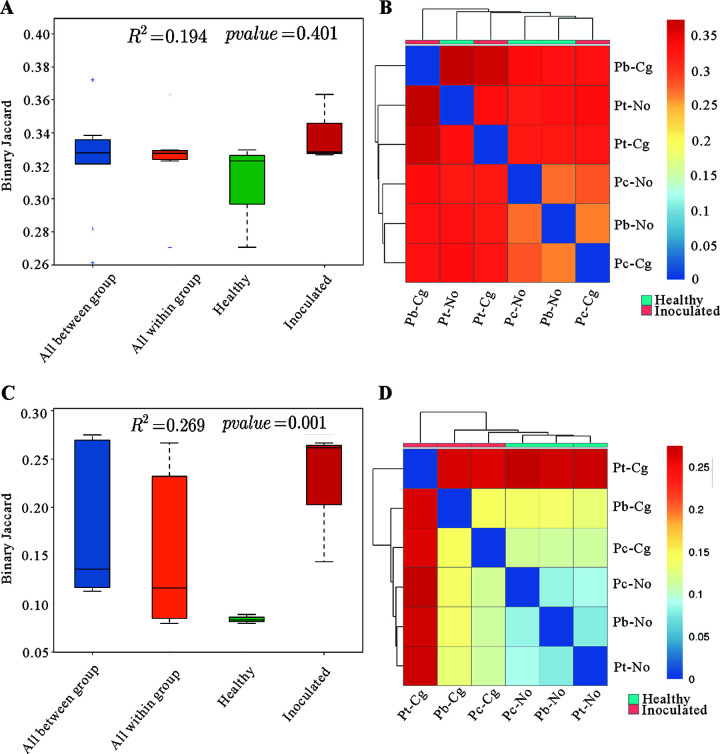
Difference analysis of phyllosphere microorganisms in healthy and susceptible poplars. (A) Bacterial PERMANOVA analysis; (B) heat map after bacterial clustering; (C) fungal PERMANOVA analysis; (D) heat map after fungal clustering. The color gradient from blue to red indicates the distance between samples from near to far, respectively. Pc-No, Pb-No, and Pt-No represent healthy *Populus* × *canadensis*, *P.* × *beijingensis*, and *P. tomentosa*, respectively; and Pc-Cg, Pb-Cg, and Pt-Cg represent *P.* × *canadensis*, *P.* × *beijingensis*, and *P. tomentosa* inoculated with *Colletotrichum gloeosporioides*, respectively.

The three species of poplar had different numbers and types of bacteria and fungi at the phylum, class, order, genus, and species level before and after inoculation. There was no significant difference between and within groups of phyllosphere bacteria in healthy poplars (HP) and inoculated poplars (IP) (permutational multivariate analysis of variance [PERMANOVA], *R*^2^ = 0.194, *P* value = 0.401) (Fig. 1A). The heat map was based on a binary Jaccard index to obtain the distance matrix between poplar samples. As shown in the heat map in Fig. 1B, there was overlap in phyllosphere bacteria between HP and IP, and the distance matrix from near to far was Pb-Cg, Pt-No, Pt-Cg, Pc-No, Pb-No, and Pc-Cg, respectively.

When samples were sorted by HP and IP, phyllosphere fungi showed greater differences between groups than within groups (PERMANOVA, *R*^2^ = 0.269, *P* value = 0.001) (Fig. 1C). A heat map was based on a binary Jaccard index to obtain the distance matrix between poplar samples. As shown on the heat map in Fig. 1D, the phyllosphere fungi differed obviously between HP and IP, and the distance matrix from near to far was Pc-Cg, Pb-Cg, Pt-Cg, Pc-No, Pb-No, and Pt-No, respectively.

### Unique and shared OTUs and dominant genera.

**FIG 2:**
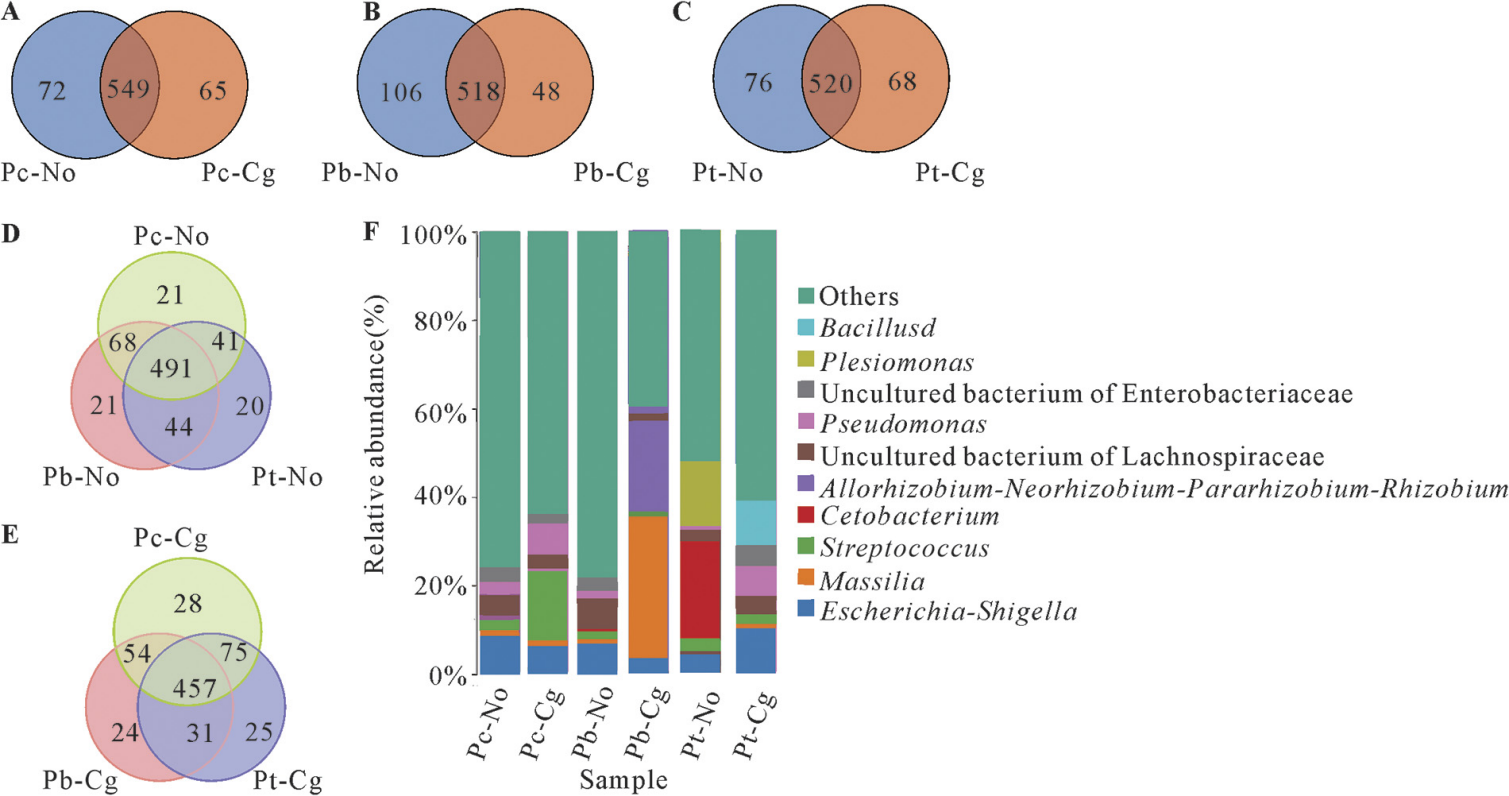
OTUs and dominant bacterial genera of three poplar species. (A) Venn diagram of *Populus* × *canadensis* before and after inoculation; (B) Venn diagram of *P.* × *beijingensis* before and after inoculation; (C) Venn diagram of *P. tomentosa* before and after inoculation; (D) Venn diagram of three healthy poplar species; (E) Venn diagram of three inoculated poplar species; (F) histogram of bacterial genus distribution of three poplar species before and after inoculation. Pc-No, Pb-No, and Pt-No represent healthy *Populus* × *canadensis*, *P.* × *beijingensis*, and *P. tomentosa*, respectively; Pc-Cg, Pb-Cg, and Pt-Cg represent *P.* × *canadensis*, *P.* × *beijingensis*, and *P. tomentosa* inoculated with *Colletotrichum gloeosporioides*, respectively.

At the genus level, same bacterial genera of the same tree species before and after inoculation accounted for more than 80% of the total bacteria. There were 491 of the same genera in healthy poplars and 457 of the same genera after inoculation, while the number of endemic genera increased after inoculation. Before and after inoculation of three species of poplar, the 10 most abundant bacterial genera were *Bacillus*, *Plesiomonas*, uncultured bacterium of *Enterobacteriaceae*, Pseudomonas, uncultured bacterium of *Lachnospiraceae*, *Rhizobium*, *Cetobacterium*, Streptococcus, *Massilia*, and *Shigella*. Although the bacterial species were similar, there were differences in bacterial abundance. After inoculation, the proportions of Pseudomonas in Pc-Cg and Pt-Cg, *Bacillus* in Pt-Cg, and *Massilia* in Pb-Cg increased (Fig. 2; Table S3).

**FIG 3:**
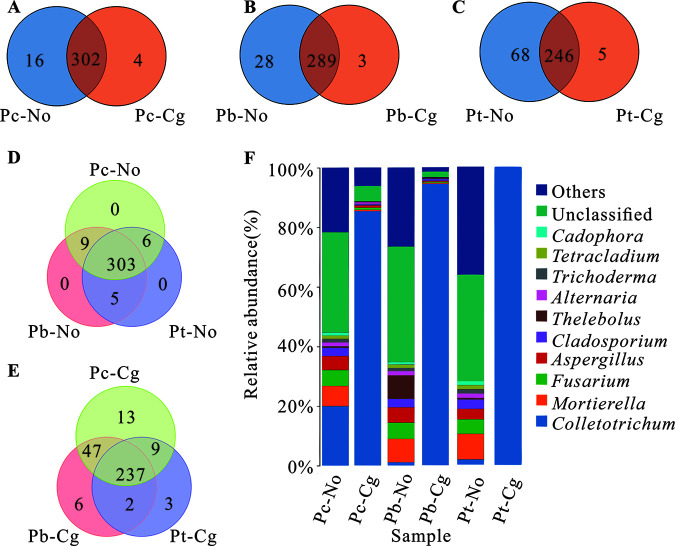
OTUs and dominant fungal genera of three poplar species. (A) Venn diagram of *Populus* × *canadensis* before and after inoculation; (B) Venn diagram of *P.* × *beijingensis* before and after inoculation; (C) Venn diagram of *P. tomentosa* before and after inoculation; (D) Venn diagram of three healthy poplar species; (E) Venn diagram of three inoculated poplar species; (F) histogram of fungal genus distribution of three poplar species before and after inoculation. Pc-No, Pb-No, and Pt-No represent healthy *Populus* × *canadensis*, *P.* × *beijingensis*, and *P. tomentosa*, respectively; Pc-Cg, Pb-Cg, and Pt-Cg represent *P.* × *canadensis*, *P.* × *beijingensis*, and *P. tomentosa* inoculated with *Colletotrichum gloeosporioides*, respectively.

Similarly, the shared fungi among poplars of the same species before and after inoculation accounted for more than 75% of the total at the genus level. Among fungal communities, Pt-No and Pt-Cg changed the most, with 68 endemic genera present before inoculation but only 5 after inoculation. The compositions of phyllosphere microbes of the three species of poplar before inoculation were similar, with 303 of the same genera and no unique genera. After inoculation, the same genera of the three species of poplar decreased to 237, while the number of endemic genera increased. Among these, those of Pc-Cg, Pb-Cg, and Pt-Cg increased to 13, 6, and 3, respectively. The number of endemic genera was positively correlated with poplar resistance. Before inoculation, the 10 most abundant fungal genera of the three poplar species were *Cadophora*, *Tetracladium*, *Trichoderma*, *Alternaria*, *Thelebolus*, *Cladosporium*, Aspergillus, Fusarium, *Mortierella*, and *Colletotrichum*, while after inoculation, the pathogen *Colletotrichum* was dominant (Fig. 3; Table S4).

### Characteristics of functional distribution of phyllosphere microorganisms.

PICRUSt2 software was used to analyze the function of bacterial 16S rRNA microbiota at different growth stages. The sequencing data were aligned using COG (Clusters of Orthologous Groups of proteins), a prokaryotic homologous protein cluster database. The results showed that the phyllosphere bacterial functions were similar before and after inoculation of the three species of poplar. The main functions were general function prediction only, amino acid transport and metabolism, carbohydrate transport and metabolism, energy production and conversion, coenzyme transport and metabolism, and cell wall/membrane/envelope biogenesis (Fig. S1 to S3).

The FUNGuild (Fungi Functional Guild) was used to analyze the fungal functions. Fungi are divided into three categories according to their nutritional methods: pathotrophs, symbiotrophs, and saprotrophs. Before inoculation, pathogenic fungi, symbiotic fungi, and saprophytic fungi were present in all three species of poplar in similar proportions; however, after inoculation, pathogenic fungi dominated (Fig. S4).

### Characteristics and differences of secondary metabolites in leaves.

**FIG 4:**
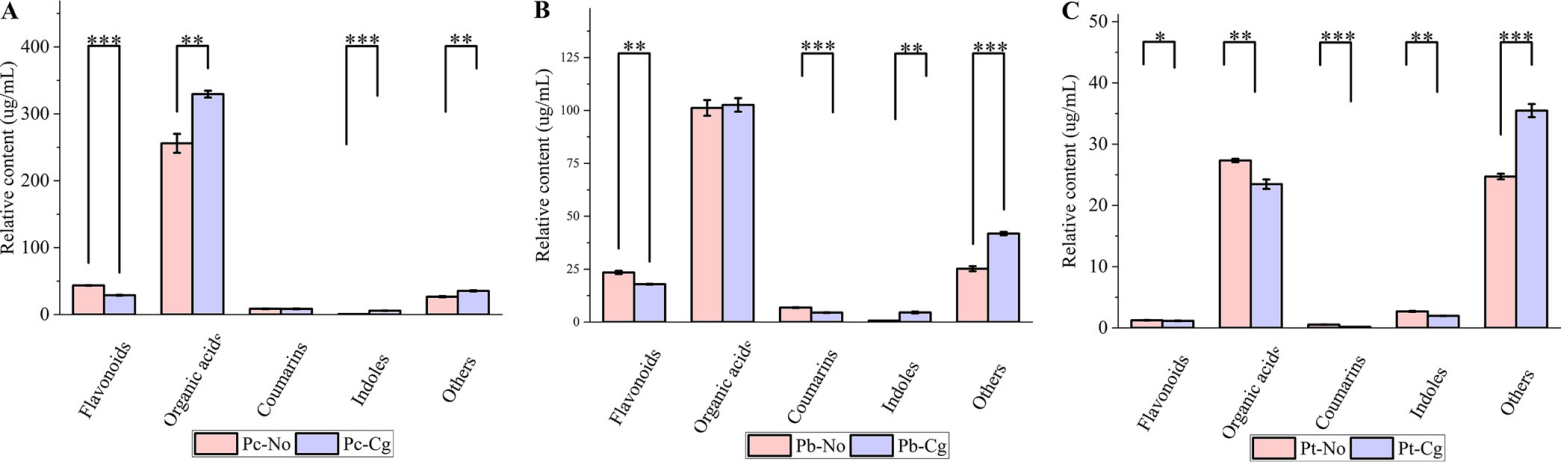
Contents of secondary metabolites before and after inoculation of three poplar species. (A) *Populus* × *canadensis*; (B) *P.* × *beijingensis*; (C) *P. tomentosa.* *, *P < *0.05; **, *P < *0.01; ***, *P < *0.001. Pc-No, Pb-No, and Pt-No represent healthy *Populus* × *canadensis*, *P.* × *beijingensis*, and *P. tomentosa*, respectively; Pc-Cg, Pb-Cg, and Pt-Cg represent *P.* × *canadensis*, *P.* × *beijingensis*, and *P. tomentosa* inoculated with *Colletotrichum gloeosporioides*, respectively.

Based on high-performance liquid chromatography–tandem mass spectrometry (HPLC-MS/MS) analysis, the secondary metabolites in the three species of poplar were divided into five categories: flavonoids, organic acids, coumarins, indoles, and others. Among the three species of poplar, the organic acid content was highest. Specifically, the organic acid level in Pc-No was 255.90 ± 14.19 μg/mL, while it increased to 329.40 ± 5.15 μg/mL in Pc-Cg. For Pb-No, this level was 101.18 ± 3.71 μg/mL, while it was 102.60 ± 3.20 μg/mL in Pb-Cg. For Pt-No, the level was 27.33 ± 0.27 μg/mL, but this decreased to 23.47 ± 0.78 μg/mL in Pt-Cg. The contents of other secondary metabolites are lower than that of organic acids in three poplar species (Fig. 4; Table 1).

**TABLE 1:** Concentration of secondary metabolites in three poplar species

Compounds	Concn (μg/mL)^*a*^ in:					
	Pc-No	Pc-Cg	Pb-No	Pb-Cg	Pt-No	Pt-Cg
Flavonoids	43.45 ± 0.34	28.79 ± 0.61	23.42 ± 0.80	17.85 ± 0.18	1.23 ± 0.03	1.14 ± 0.04
Organic acids	255.90 ± 14.19	329.40 ± 5.15	101.18 ± 3.71	102.60 ± 3.20	27.33 ± 0.27	23.47 ± 0.78
Coumarins	8.58 ± 0.39	8.47 ± 0.50	6.83 ± 0.17	4.42 ± 0.12	0.54 ± 0.02	0.18 ± 0.01
Indoles	0.77 ± 0.04	5.73 ± 0.32	0.62 ± 0.02	4.48 ± 0.42	2.70 ± 0.07	1.96 ± 0.02
Others	26.59 ± 0.83	35.36 ± 0.95	25.18 ± 1.18	41.79 ± 0.82	24.71 ± 0.45	35.48 ± 1.07

a Pc-No, Pb-No, and Pt-No represent healthy *Populus* × *canadensis*, *P.* × *beijingensis*, and *P. tomentosa*, respectively; Pc-Cg, Pb-Cg, and Pt-Cg represent *P.* × *canadensis*, *P.* × *beijingensis*, and *P. tomentosa* inoculated with *Colletotrichum gloeosporioides*, respectively.

### Relationship between leaf secondary metabolites and microorganisms.

**FIG 5:**
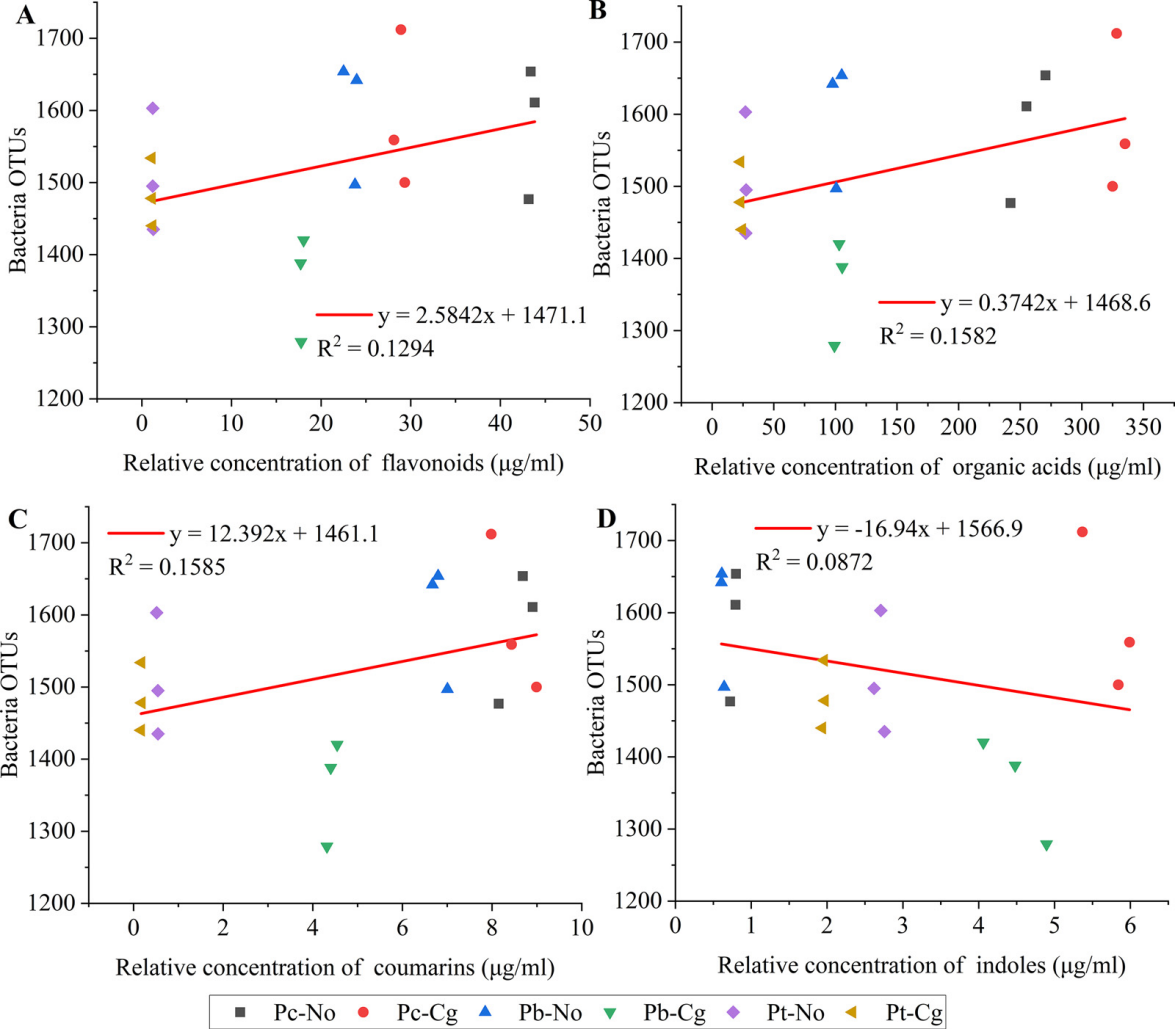
Regression equations between the content of four important secondary metabolites and the number of bacteria. The values on the abscissa are the relative content of the substances, and those on the ordinate are the number of bacterial OTUs. (A) Flavonoids; (B) organic acids; (C) coumarins; (D) indoles. Pc-No, Pb-No, and Pt-No represent healthy *Populus* × *canadensis*, *P.* × *beijingensis*, and *P. tomentosa*, respectively; Pc-Cg, Pb-Cg, and Pt-Cg represent *P.* × *canadensis*, *P.* × *beijingensis*, and *P. tomentosa* inoculated with *Colletotrichum gloeosporioides*, respectively.

**FIG 6:**
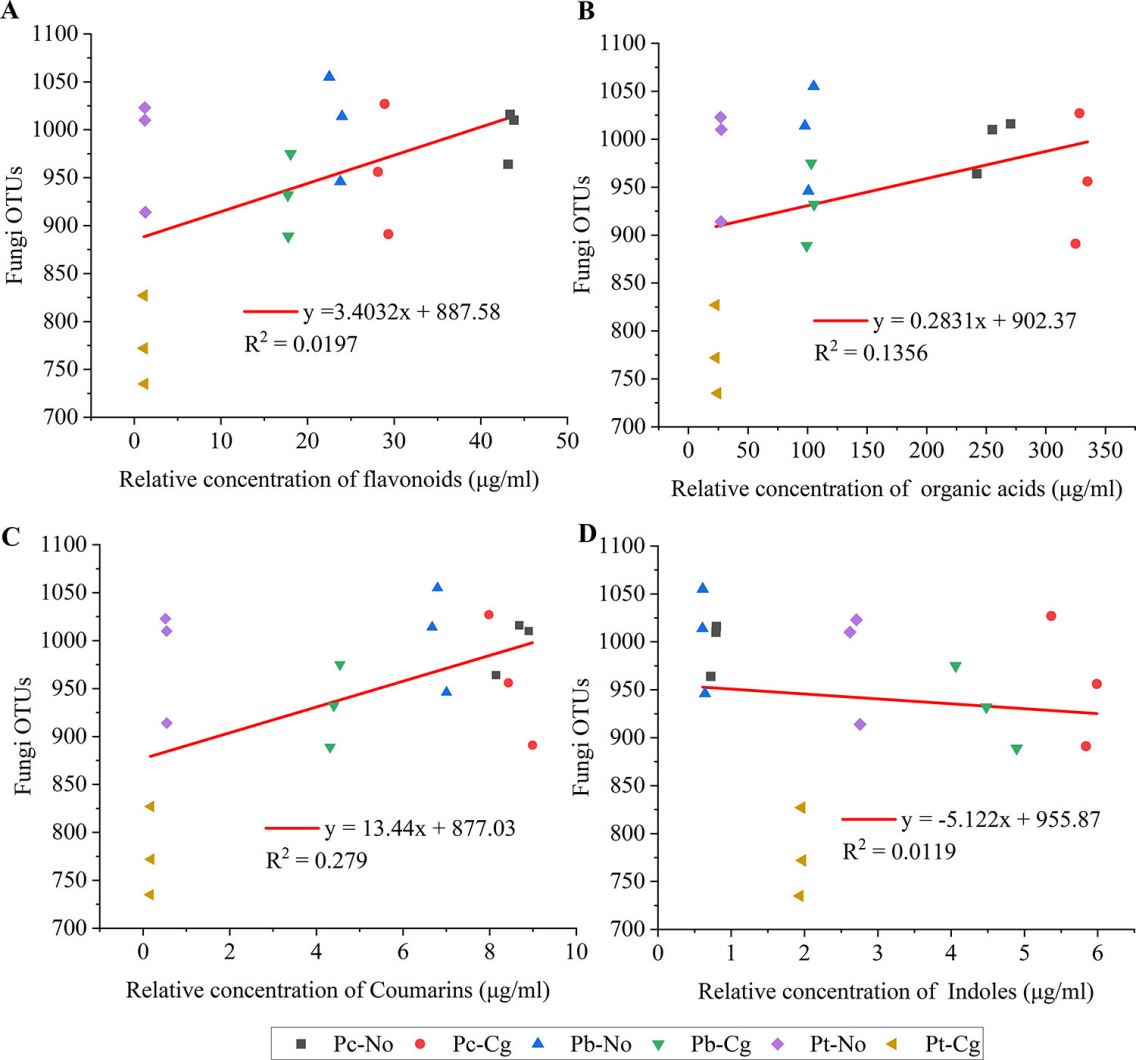
Regression equations between the content of four important secondary metabolites and the number of fungi. The values on the abscissa are the relative content of the substances, and those on the ordinate are the number of fungal OTUs. (A) Flavonoids; (B) organic acids; (C) coumarins; (D) indoles. Pc-No, Pb-No, and Pt-No represent healthy *Populus* × *canadensis*, *P.* × *beijingensis*, and *P. tomentosa*, respectively; Pc-Cg, Pb-Cg, and Pt-Cg represent *P.* × *canadensis*, *P.* × *beijingensis*, and *P. tomentosa* inoculated with *Colletotrichum gloeosporioides*, respectively.

Regression analyses were conducted using flavonoids, organic acids, coumarins, and indoles as the abscissa and the numbers of phyllosphere bacteria and phyllosphere fungi as the ordinates for the three poplar species before and after inoculation. For bacteria, coumarins had the best fit (*R*^2^ = 0.1585), indicating that they had the greatest effect on phyllosphere bacteria. This was followed by organic acids (*R*^2^ = 0.1582), flavonoids (*R*^2^ = 0.1294), and indoles (*R*^2^ = 0.0872) (Fig. 5). For fungi, coumarins had the best fit (*R*^2^ = 0.2790), indicating that coumarins had the greatest effect on phyllosphere fungi. This was followed by organic acids (*R*^2^ = 0.1356), flavonoids (*R*^2^ = 0.0197), and indoles (*R*^2^ = 0.0119) (Fig. 6).

Increases in coumarins, flavonoids, and organic acids were correlated with increases in the number of microorganisms, suggesting that they had a recruiting effect on microorganisms. Conversely, the number of microorganisms decreased with increasing concentrations of indoles, suggesting that they had inhibitory effects on microorganisms. In addition, *R*^2^ values are low and *P* values are greater than 0.01 in the regression equations. So, it is considered that the secondary metabolites have influences on phyllosphere microorganisms in poplar leaves, but they are not the main influence (Fig. 5 and 6; Table S5).

## DISCUSSION

Plants contain a variety of bacteria and fungi that play positive or negative roles in the pathogenesis of phytopathogens (22, 23). Both the aerial and underground parts of plants contain a large number of microorganisms; however, research on phyllosphere microbial communities has long lagged behind that on rhizosphere microbial communities (12, 13). In this study, poplar anthracnose, an important poplar leaf disease, was used to explore differences in phyllosphere microbial communities before and after inoculation with *C. gloeosporioides* of three poplar species with different resistances.

The compositions of phyllosphere microbes of the three poplar species before inoculation were similar, and most bacteria were shared among poplar species, although there were some differences (Fig. 2). Simultaneously, we found that the resistance of different poplar species was not related to the number of bacteria, so we speculated that the resistance gradient was related to the key bacterial genera. We focused on three bacteria that differed greatly before and after *C. gloeosporioides* inoculation, indicating they may be related to resistance: Pseudomonas, *Bacillus*, and *Massilia*. The proportion of Pseudomonas bacteria in Pc-No is higher than that in Pb-No and the lowest in Pt-No, which is positively related to their resistance to *C. gloeosporioides*. In addition, the proportion of Pseudomonas in Pc-Cg and Pt-Cg increased after inoculation (Fig. 2F; see Table S3 in the supplemental material). Pseudomonas is an important biocontrol bacterium that can effectively inhibit Fusarium *culmorum* (24), the oomycete *Pythium* (25), Fusarium oxysporum, Geotrichum candidum (26), and other phytopathogenic fungi. Additionally, the proportion of *Bacillus* in Pt-Cg increased (Fig. 2F; Table S3). Backman and Sikora found that Bacillus mojavensis, which had strong viability in *Coffea* spp., exerted a broad spectrum of antifungal activity (27). Collins et al. studied the control effect of Bacillus subtilis application on sugar beet *Cercospora* leaf spot (28). The proportion of *Massilia* in Pb-Cg also increased (Fig. 2F; Table S3). *Massilia* is a potential biocontrol bacterium that has been used to prevent *Rhizoctonia* infestation without damaging the soils or affecting bacterial and fungal biodiversity (29). Based on the results of the present and previous studies, additional investigations to confirm whether Pseudomonas, *Bacillus*, and *Massilia* can inhibit *C. gloeosporioides* are warranted.

In addition to the bacteria, previous studies have shown that fungi have antagonistic effects on *C. gloeosporioides*. For example, Cryptococcus laurentii controls mango anthracnose by forming biofilms on mango fruits and competing with *C. gloeosporioides* for nutrients (30). Meyerozyma caribbica can colonize on the surface of fruits, thereby competing with *C. gloeosporioides* for space while also producing a variety of fungus-inhibiting enzymes, which makes it useful for preventing and treating anthracnose (31). However, because of the typical characteristics of latent *Colletotrichum* inoculation, some biocontrol factors cannot be used after the pathogen invades the host (32). Therefore, currently available prevention and control methods focus mainly on preventing the occurrence of diseases, which depends on the balance of phyllosphere microbial communities (33).

Plant secondary metabolites influence microbiota composition and function and play an important role in shaping microbial community assemblages, and plant secondary metabolites were altered by fungal infections (34–36). In this study, HPLC-MS/MS revealed that the content of secondary metabolites in the three poplar species was positively related to their resistance to *C. gloeosporioides* (Fig. 4), which may explain the effect of the secondary metabolites on the microbial community, making different poplar species have differing resistances to *C. gloeosporioides*. Moreover, regression analysis revealed that coumarin had the greatest impact on the number of microorganisms in the three poplar species, followed by flavonoids, organic acids, and indoles. In addition, the slopes of the coumarin, flavonoid, and organic acid regression curves were greater than 1, while that of indoles was less than 1. Therefore, these findings suggest that coumarin, flavonoids, and organic acids have recruitment effects on microorganisms, while indoles have inhibitory effects on microorganisms (Fig. 5 and 6).

Coumarin can affect microbial community composition, possibly through its impact on soil iron availability. Coumarin secreted by Arabidopsis thaliana roots can rebuild the structure of the rhizosphere microbial community, thus promoting plant growth (37). Therefore, it will be possible to enhance plant iron nutrition through coumarin-mediated changes in the rhizosphere microbiome (38). Flavonoids can also improve soil nutrient activity by modulating the root microbiome and affecting microbial interactions with plants (39). As a result, flavonoids are considered to be crucial root-released rhizosphere signaling molecules that regulate root-microbe interactions (39, 40). Flavonoids can also affect crop growth as auxin transport regulators (41, 42), indicating that flavonoid-mediated plant-microbe interactions may also be involved in processes that regulate host plant development (40, 43). Fatty acids and aromatic acids in organic acids can be used as nutrient carbon and nitrogen sources to stimulate the growth of microorganisms (44, 45). In addition, organic acids can induce signals to promote the biological activity of soil rhizosphere microorganisms (46). Indole and its derivatives can be used to synthesize benzoxazinoids, and benzoxazinoids mainly play a role in plant resistance to insects, which can be secreted from roots as allelopathic substances to inhibit the reproduction of pathogenic bacteria. A previous study of the effects of the Zea mays root exudate benzoxazolinone on a rhizosphere microbial community revealed that it mediated the rhizosphere microbial community through root secondary metabolites (47). Additionally, synthetic mutants of benzoxazinoids were found to alter rhizosphere bacterial and fungal communities as well as to have the potential to act as part of a plant-soil feedback mechanism linking changes in the microbiota to disease resistance in progeny plants (48, 49).

We also found that there were a large number of *Colletotrichum* fungi in *P. × canadensis* before inoculation (Fig. 3F). However, the species present were Colletotrichum jasminigenum and Colletotrichum graminicola not the pathogen *C. gloeosporioides*. These fungi are not known to be pathogenic fungi responsible for poplar anthracnose; therefore, we speculated that they may produce antibodies that impart an immune effect to *P. × canadensis* against poplar anthracnose. If so, *C. jasminigenum* and *C. graminicola* could have a strong immune effect on poplar anthracnose resistance. Accordingly, subsequent experiments will be conducted to investigate this.

In conclusion, this study investigated the effects of *Colletotrichum gloeosporioides* and poplar secondary metabolites on the composition of poplar phyllosphere microbial communities. We selected the important microbial genera that may have biocontrol effects on poplar anthracnose and the key secondary metabolites that may recruit or inhibit the phyllosphere microorganisms, which will be verified in future research. This study provides information that can be used to develop novel methods of poplar anthracnose control.

## MATERIALS AND METHODS

### Plant and fungal materials.

Annual branches of *P. × canadensis* (Pc), *P. × beijingensis* (Pb), and *P. tomentosa* (Pt) collected from Yanqing District, Beijing, China (N40°28′, E115°50′), were hydroponically cultivated with sterile water for 1 month at 25 ± 2°C, 60% to 70% relative humidity, under a 16-h light (2,000 lx)/8-h dark photoperiod to make them grow new leaves. The strain *Colletotrichum gloeosporioides* CFCC 80308 (Cg) was provided by the Laboratory of Forest Pathology, Beijing Forestry University. After culture of *C. gloeosporioides* on potato dextrose agar (PDA) medium for 7 days, the medium surface was repeatedly flooded with sterile water, and the spore fluid concentration was adjusted to 10^6^/mL. Then, 30 μL of conidial suspension was inoculated onto the new leaves of the three species of poplar materials (Pc-Cg, Pb-Cg, and Pt-Cg) as the experimental group. Similarly, 30 μL of sterile water was dripped onto the leaves of three species of poplar (Pc-No, Pb-No, and Pt-No) as control groups. The leaves of three species of poplar in the experimental groups and the control groups were cultured for 6 days under the above-described conditions (see Fig. S5 in the supplemental material).

### Extraction of total DNA from samples.

Ten grams of leaves of three poplar species in the experimental group and the control group were cut into scraps, placed into a sterile conical flask, and then diluted 1:20 (ratio of leaf weight to volume of TE buffer) with sterile TE buffer (10 mmol/L Tris-HCl, 1 mmol/L EDTA, pH 8.0). The tube was then sealed with sterilization film, shaken at room temperature for 30 min on a shaker at 200 rpm to separate the microbial cells from the leaf surface, and then sonicated at 40 kHz for 15 min. Microorganisms in the shaking solution were subsequently collected on a 0.22-μm filter using a vacuum filtration device in a sterile environment, after which the total DNA on the filter was extracted using a FastDNA spin kit (Qbiogene, Irvine, CA, USA) according to the kit instructions. The extracted DNA was then dissolved in 100 μL of double-distilled water and stored at −20°C until subsequent analysis (50).

### MiSeq high-throughput sequencing.

The high-throughput Illumina MiSeq sequencing platform was used to sequence the V4 region of the bacterial 16S rRNA gene and the ITS1 region of the fungal ITS gene. The total number of bacterial and fungal sequences was 96. The bacterial primers were 515F (GTGCCAGCMGCCGCGGTAA) and 907R (CCGTCAATTCMTTTRAGTTT), and the fungal primers were ITS-5F (GGAAGTAAAAGTCGTAACAAGG) and ITS-1R (GCTGCGTTCTTCATCGATGC). The PCR amplification products were detected by 2% agarose gel electrophoresis, after which they were subjected to fluorescence quantification. The products were then subjected to sequence and analyze (https://www.biocloud.net).

### Processing of sequencing data.

Quality filtering of the raw tags was performed under specific filtering conditions to obtain high-quality clean tags according to QIIME software (Quantitative Insights Into Microbial Ecology; v1.8.0). The UCHIME method in Mothur software was then used to remove chimeric sequences and chloroplast-derived and mitochondrial origin sequences. Hence, only high-quality sequences were used for experimental analysis. The UCLUST sequence alignment tool in the QIIME software was then used to obtain OTUs with 97% sequence similarity, which were subsequently searched by BLAST against bacterial or fungal databases for identification and to obtain taxonomic information (50). Specifically, bacterial sequences were submitted to the Greengenes and Silva databases, while fungal sequences were compared to the fungal UNITE database and the Silva database.

### Extraction and determination of secondary metabolites.

The samples were kept at −40°C for 24 h and then stored in an ultra-low-temperature freezer (51). Frozen samples were ground to a powder (30 Hz, 1 min) using a MM 400 grinder (Retsch, Germany) and then stored at −20°C until analysis. For analysis, 10 μL of the internal standard 2-amino-3-(2-chloro-phenyl)-propionic acid (CAS no. 14091-11-3) was added to 200 mg of ground sample to give a final concentration of 100 ppm. Samples were then subjected to methanol treatment with an extraction solution consisting of methanol-water-formic acid (15:4:1, vol/vol/vol). After centrifugation in a centrifuge (Jouan BR4i, Thermo Fisher Scientific, USA) at 10,000 × *g* for 3 min at −4°C, the extracts were dried with nitrogen gas and then reconstituted with 100 μL of 80% acetonitrile-water solution, filtered through a 0.22-μm polytetrafluoroethylene membrane, and centrifuged.

A 10 μL-aliquot of each sample was subjected to HPLC-MS/MS analysis. Chromatographic separations were performed at 35°C using a Zorbax Eclipse C_18_ column (2.1 by 100 mm, 3 μm [inner diameter]) (Agilent Technologies, USA). The mobile phase consisted of 95% solution A (5 mM ammonium acetate in water containing 0.1% formic acid) and 5% methanol. The flow rate was 0.3 mL/min, and the injection volume was 10 μL. The type and relative content of the compounds were determined using HPLC-MS/MS (52).

### Data analysis.

The alpha diversity was calculated using Mothur (v.1.42.0) software. Specifically, the Shannon diversity index (https://mothur.org/wiki/shannon/) and Simpson diversity index (https://mothur.org/wiki/simpson/) were calculated. PICRUSt2 software (https://github.com/picrust/picrust2/) was used to annotate the species between the characteristic sequences to be predicted and the existing phylogenetic trees in the software, and the microbial genome data of IMG were used to output functional information and then speculate as to the composition of functional genes in the samples, so as to analyze the functional differences between different samples or groups. FUNGuild (http://www.funguild.org/) was used to parse fungal functions through ecological association taxonomy using a simple and consistent method to classify large sequence libraries into ecologically significant classes. PERMANOVA was performed using R software (www.r-project.org/). Statistical analyses were performed using SPSS 17.0 (SPSS, Inc., Chicago, IL, USA) and Origin 8.0 (OriginLab). One-way analysis of variance followed by Duncan’s test was used to determine the statistical significance of differences among treatments (53).
