# Supplementary material for: Effects of Colletotrichum gloeosporioides and Poplar Secondary Metabolites on the Composition of Poplar Phyllosphere Microbial Communities
Source: Microbiol Spectr. 2023 May 23;11(3):e04603-22. doi: 10.1128/spectrum.04603-22 (PMC10269685; doi:10.1128/spectrum.04603-22)
Supplement: Supplemental file 1 — Fig. S1 to S5, Tables S1 to S5. Download spectrum.04603-22-s0001.pdf, PDF file, 1.1 MB [file spectrum.04603-22-s0001.pdf]

Supplementary Fig. 1 Statistical map of COG functional classification of bacteria in *Populus* × *canadensis.*

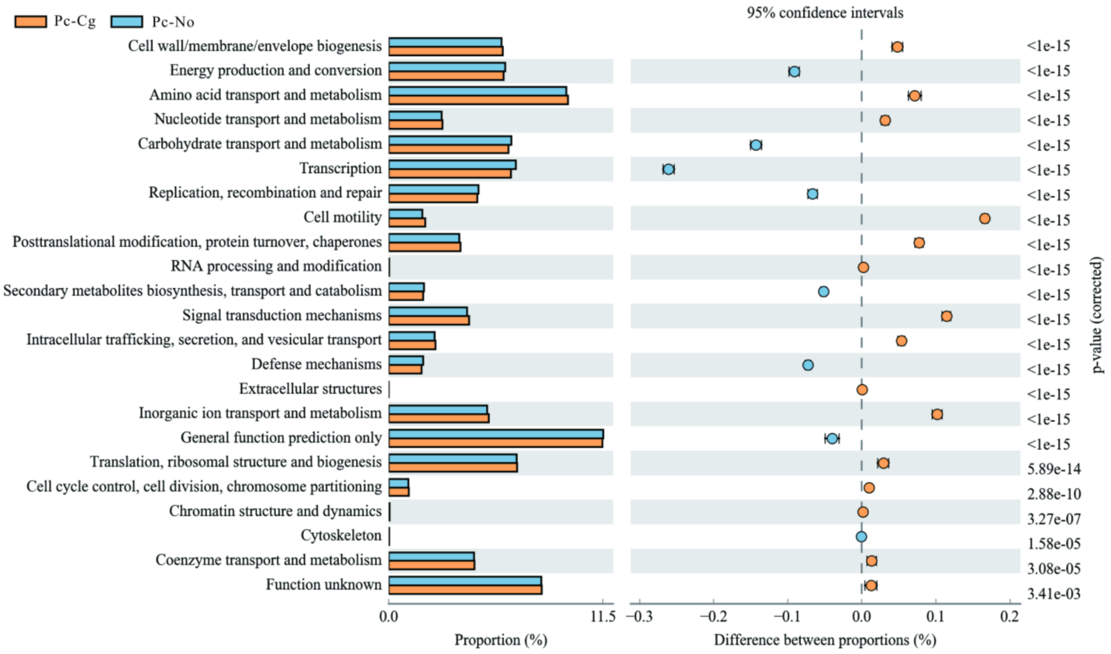

Pc-No = healthy *Populus* × *canadensis* and Pc-Cg = *P.* × *canadensis* inoculated with *Colletotrichum gloeosporioides*.

Supplementary Fig. 2 Statistical map of COG functional classification of bacteria in *P.* × *beijingensis*.

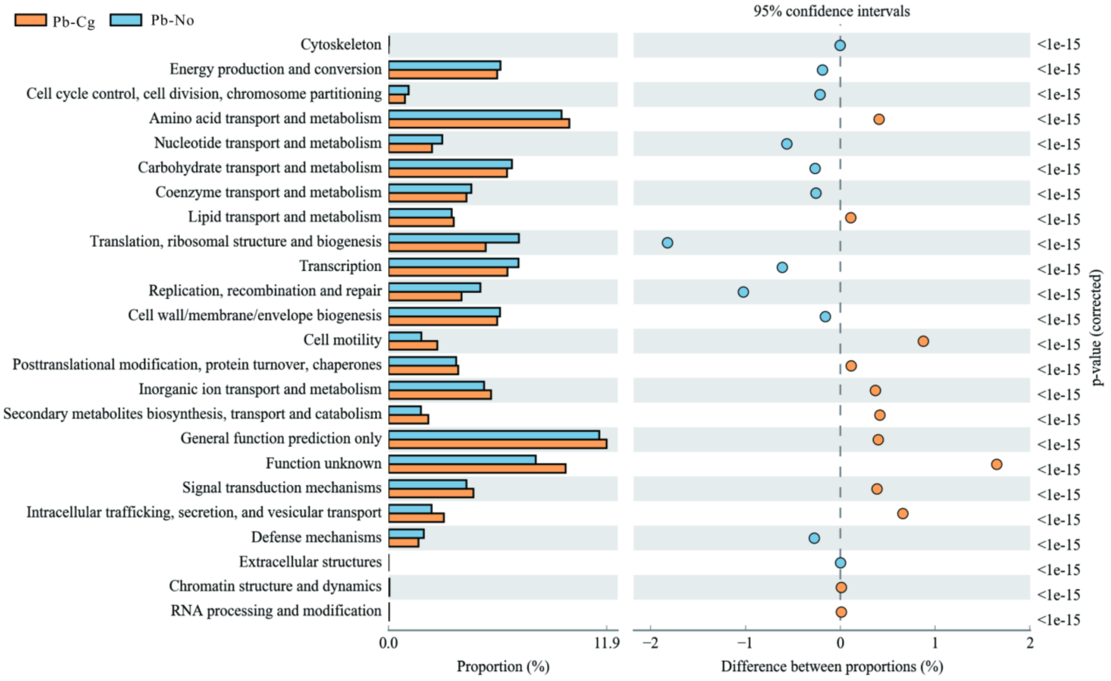

Pb-No = healthy *P.* × *beijingensis* and Pb-Cg = *P.* × *beijingensis* inoculated with *Colletotrichum gloeosporioides*.

Supplementary Fig. 3 Statistical map of COG functional classification of bacteria in *P.tomentos*.

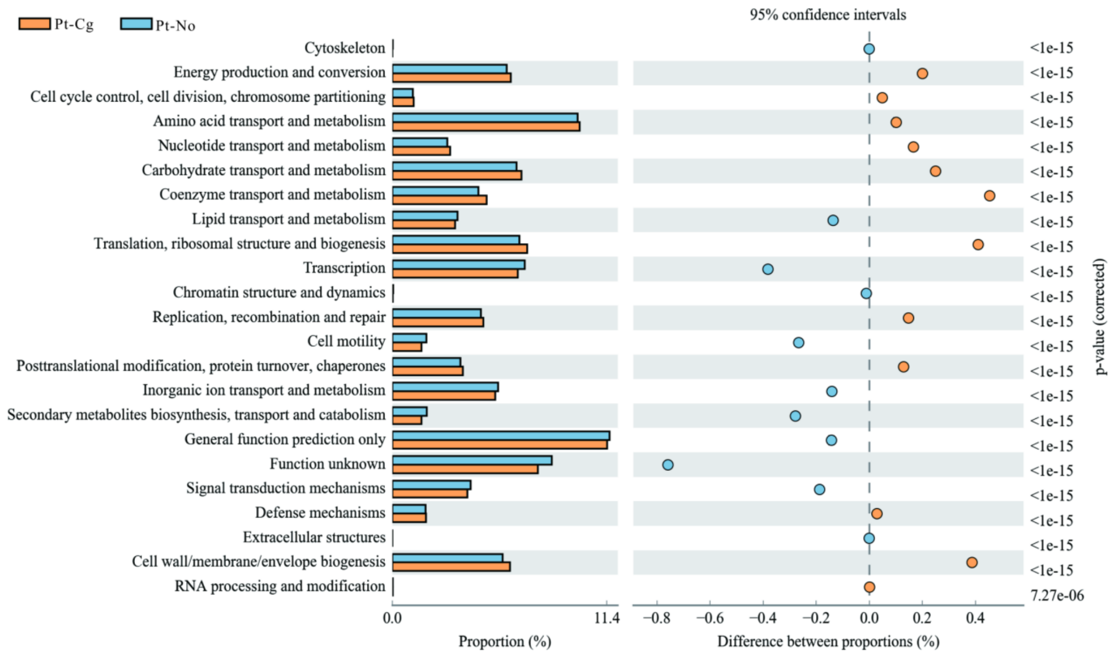

Pt-No = healthy *P. tomentosa* and Pt-Cg = *P. tomentosa* inoculated with *Colletotrichum gloeosporioides*.

Supplementary Fig. 4 Funguild histogram of fungi in three species of poplars before and after inoculation.

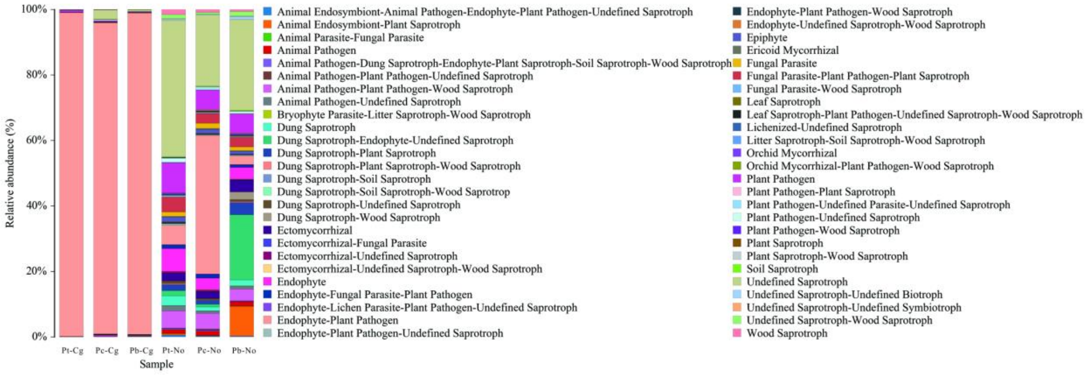

Pc-No, Pb-No and Pt-No = healthy *Populus* × *canadensis*, *P.* × *beijingensis* and *P. tomentosa*, respectively; and Pc-Cg, Pb-Cg and Pt-Cg = *P.* × *canadensis*, *P.* × *beijingensis* and *P. tomentosa* inoculated with *Colletotrichum gloeosporioides*, respectively.

Supplementary Fig. 5 Differences in the resistance of three poplar species to poplar anthracnose. Anthracnose lesions appeared on leaves of three types of poplar in 6 dpi (Days Post Inoculation) with *C. gloeosporioides*.

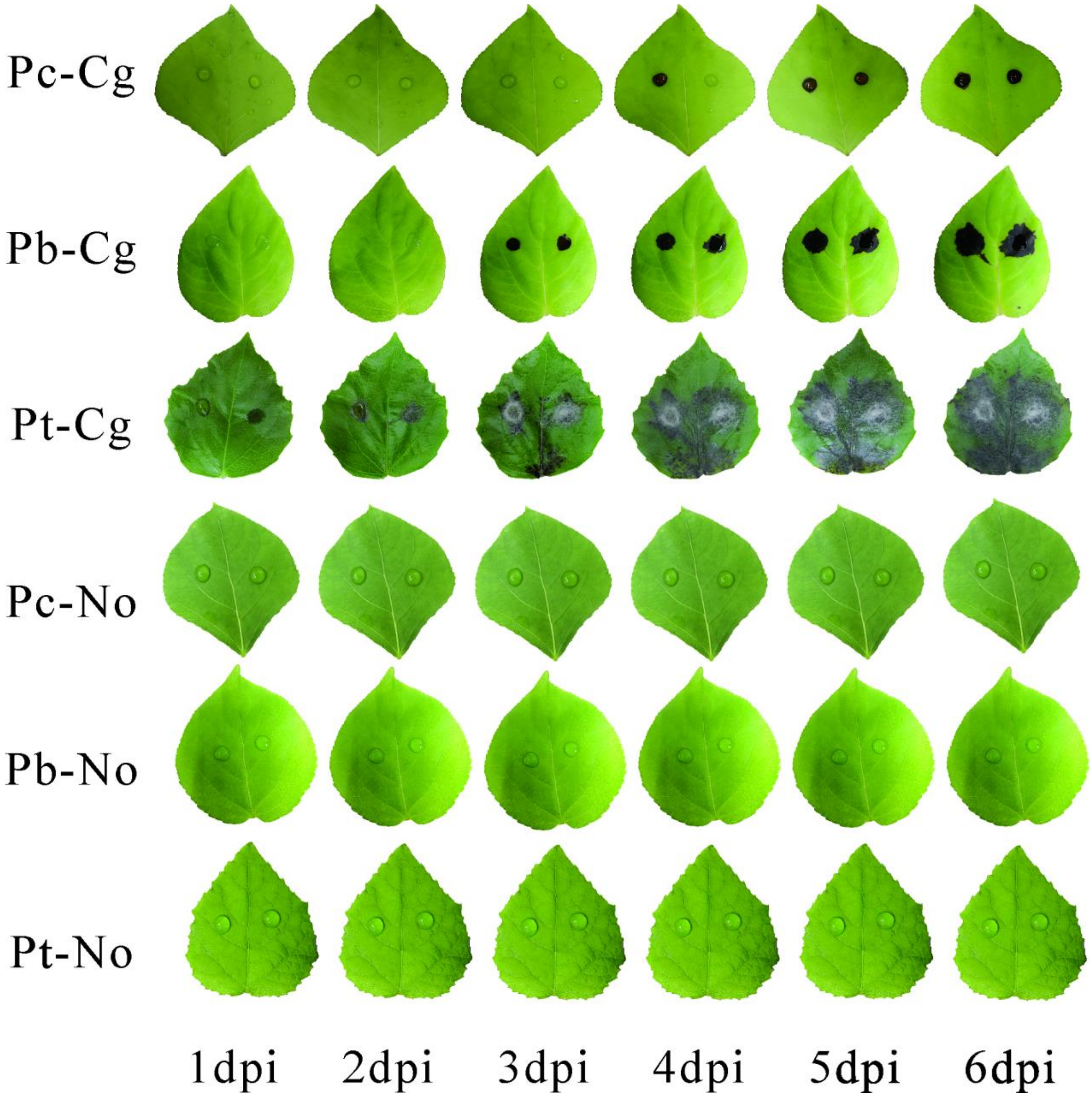

Pc-No, Pb-No and Pt-No = healthy *Populus* × *canadensis*, *P.* × *beijingensis* and *P. tomentosa*, respectively; and Pc-Cg, Pb-Cg and Pt-Cg = *P.* × *canadensis*, *P.* × *beijingensis* and *P. tomentosa* inoculated with *Colletotrichum gloeosporioides*, respectively.

Supplementary Table 1. Statistical table for evaluating the Alpha diversity index of phyllosphere bacteria.

Sample	OTUs	Simpson	Shannon
Pc-No	1612.67 ± 92.32	0.99 ± 0.01	9.06 ± 0.84
Pc-Cg	1590.33 ± 109.42	0.97 ± 0.02	8.26 ± 0.93
Pb-No	1597.67 ± 87.39	0.99 ± 0.01	9.20 ± 0.79
Pb-Cg	1362.33 ± 73.92	0.87 ± 0.03	5.64 ± 1.05
Pt-No	1511.00 ± 85.14	0.93 ± 0.02	6.97 ± 1.14
Pt-Cg	1484.00 ± 47.29	0.97 ± 0.01	8.01 ± 1.26

Shannon, Simpson, represent each index respectively. Pc-No, Pb-No and Pt-No = healthy *Populus × canadensis*, *P. × beijingensis* and *P. tomentosa*, respectively; and Pc-Cg, Pb-Cg and Pt-Cg = *P. × canadensis*, *P. × beijingensis* and *P. tomentosa* inoculated with *Colletotrichum gloeosporioides*, respectively.

Supplementary Table 2. Statistical table for evaluating the Alpha diversity index of phyllosphere fungi.

Sample	OTUs	Simpson	Shannon
Pc-No	996.67 ± 28.45	0.96 ± 0.02	7.60 ± 0.58
Pc-Cg	958.00 ± 68.02	0.27 ± 0.13	1.47 ± 0.23
Pb-No	1005.00 ± 55.05	0.99 ± 0.01	8.12 ± 0.62
Pb-Cg	932.00 ± 43.00	0.11 ± 0.06	0.74 ± 0.16
Pt-No	982.33 ± 59.53	0.99 ± 0.01	8.03 ± 0.74
Pt-Cg	778.00 ± 46.29	0.06 ± 0.03	0.35 ± 0.09

Shannon, Simpson represent each index respectively. Pc-No, Pb-No and Pt-No = healthy *Populus* × *canadensis*, *P.* × *beijingensis* and *P. tomentosa*, respectively; and Pc-Cg, Pb-Cg and Pt-Cg = *P.* × *canadensis*, *P.* × *beijingensis* and *P. tomentosa* inoculated with *Colletotrichum gloeosporioides*, respectively.

Supplementary Table 3. Average abundance of phyllosphere bacteria in three species of poplars before and after inoculation.

	Pb-No	Pb-Cg	Pc-No	Pc-Cg	Pt-No	Pt-Cg
Escherichia-Shigella	0.070370954	0.034464333	0.087229412	0.063076071	0.042293845	0.101697706
Massilia	0.008584705	0.321232968	0.01146477	0.012520274	0.005760329	0.009330065
Streptococcus	0.017504135	0.007814587	0.024717267	0.156750663	0.028578776	0.020763968
Cetobacterium	0.006123494	0.00138592	0.003478295	0.003322916	0.221344077	0.002597783
Allorhizobium-Neorhizobium-Pararhizobium-Rhizobium	0.004961802	0.206619022	0.00567409	0.003995411	0.003000171	0.004500384
Uncultured bacterium of Lachnospiraceae	0.063558321	0.014710793	0.046811239	0.029194193	0.02122407	0.033222348
Pseudomonas	0.018114515	0.005744056	0.029769539	0.072154753	0.008743357	0.070871904
Uncultured bacterium of Enterobacteriaceae	0.024119871	0.008883249	0.028564766	0.016416789	0.01760672	0.044180601
Plesiomonas	0.00380011	0.000751403	0.001787727	0.002867993	0.128767358	0.001445245
Bacillus	0.005414665	0.002070532	0.005168862	0.00510305	0.004097377	0.101898943
Others	0.777447429	0.396323137	0.755334033	0.634597888	0.518583919	0.609491054

Pc-No, Pb-No and Pt-No = healthy *Populus* × *canadensis*, *P.* × *beijingensis* and *P. tomentosa*, respectively; and Pc-Cg, Pb-Cg, and Pt-Cg = *P.* × *canadensis*, *P.* × *beijingensis* and *P. tomentosa* inoculated with *Colletotrichum gloeosporioides*, respectively.

Supplementary Table 4. Average abundance of phyllosphere fungi in three species of poplars before and after inoculation.

	Pb-No	Pb-Cg	Pc-No	Pc-Cg	Pt-No	Pt-Cg
Colletotrichum	0.010461506	0.944344383	0.19969302	0.853546505	0.017238234	0.970982698
Mortierella	0.079372509	0.003544157	0.067524488	0.004865071	0.086084518	0.000811662
Fusarium	0.053968128	0.003515875	0.053783898	0.00693298	0.048613391	0.001031197
Aspergillus	0.051737402	0.003919263	0.046432414	0.00770106	0.034908687	0.000746729
Cladosporium	0.028150464	0.006177342	0.028684151	0.004912338	0.031894381	0.001021921
Thelebolus	0.078832255	0.001647787	0.004078959	0.000275158	0.004762827	0.000063386
Alternaria	0.014459883	0.002219378	0.013595789	0.005869484	0.015700209	0.003579043
Trichoderma	0.010371879	0.000503118	0.011321284	0.001163093	0.013968527	0.00015924
Tetracladium	0.011873137	0.000378083	0.011029453	0.000639786	0.013365104	0.000129866
Cadophora	0.008429952	0.000232209	0.010768809	0.000162056	0.013816969	0.000055656
Others	0.38688154	0.019245025	0.336622194	0.051996502	0.357155888	0.012399103
Unclassified	0.265461344	0.014273381	0.21646554	0.061935967	0.362491264	0.009019497

Pc-No, Pb-No and Pt-No = healthy *Populus* × *canadensis*, *P.* × *beijingensis*, and *P. tomentosa*, respectively; and Pc-Cg, Pb-Cg and Pt-Cg = *P.* × *canadensis*, *P.* × *beijingensis* and *P. tomentosa* inoculated with *Colletotrichum gloeosporioides*, respectively.

Supplementary Table 5. R^2 and *p-value* in the regression equations between the four important secondary metabolites and the number of bacteria and fungi.

Microorganisms	Secondary metabolites	R^2	*p-value*
Bacteria	Coumarins	0.1585	0.10
	Organic acids	0.1582	0.10
	Flavonoids	0.1294	0.14
	Indoles	0.0872	0.23
Fungi	Coumarins	0.2790	0.03
	Organic acids	0.1356	0.13
	Flavonoids	0.0197	0.03
	Indoles	0.0119	0.67
